# Supplementary material for: Effectiveness and Safety of Ayurvedic Medicines in Type 2 Diabetes Mellitus Management: A Systematic Review and Meta-Analysis
Source: Front Pharmacol. 2022 Jun 8;13:821810. doi: 10.3389/fphar.2022.821810 (PMC9213670; doi:10.3389/fphar.2022.821810)

Study or Subgroup	Mean Difference	SE	Intervention Total	Comparator Total	Weight	Mean Difference IV, Random, 95% CI
Mahluji 2013	−0.6	1.19	28	30	2.6%	−0.60 [−2.93, 1.73]
Arablou 2014a, Arablou 2014b	0	0.87	33	30	4.9%	0.00 [−1.71, 1.71]
Azimi 2014, Azimi 2016	0.82	0.22	41	39	77.3%	0.82 [0.39, 1.25]
Mozaffari-Khosravi 2014, Talaei 2017, Talaei 2018	−0.48	0.83	40	41	5.4%	−0.48 [−2.11, 1.15]
Shidfar 2015	0	0.74	22	23	6.8%	0.00 [−1.45, 1.45]
Arzati 2017, Zarezadeh 2018	0.5	1.17	23	22	2.7%	0.50 [−1.79, 2.79]
Mohammadi 2017, Mohammadi 2019	−0.38	7.48	11	11	0.1%	−0.38 [−15.04, 14.28]
**Total (95% CI)**			**198**	**196**	**100.0%**	**0.61 [0.23, 0.98]**

Heterogeneity: $Tau^2 = 0.00$; $Chi^2 = 4.87$, df = 6 (P = 0.56); $I^2 = 0\%$

Test for overall effect: Z = 3.13 (P = 0.002)

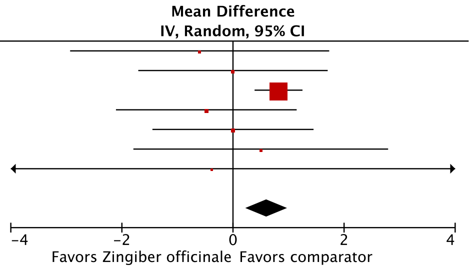

Supplement: Supplementary file 1 [file DataSheet1.zip › Supplementary Material/Forest and Funnel Plots/Zingiber officinale/BMI.pdf]
